# Nanocomposite Bioprinting for Tissue Engineering Applications

**DOI:** 10.3390/gels9020103

**Published:** 2023-01-24

**Authors:** Konstantinos Loukelis, Zina A. Helal, Antonios G. Mikos, Maria Chatzinikolaidou

**Affiliations:** 1 Department of Materials Science and Technology, University of Crete, 70013 Heraklion, Greece; 2 Department of Bioengineering, Rice University, Houston, TX 77030, USA; 3 Institute of Electronic Structure and Laser (IESL), Foundation for Research and Technology Hellas (FO.R.T.H), 70013 Heraklion, Greece

**Keywords:** composite, extrusion, 3D printing, stereolithography, inkjet, bone, cartilage, cardiovascular

## Abstract

Bioprinting aims to provide new avenues for regenerating damaged human tissues through the controlled printing of live cells and biocompatible materials that can function therapeutically. Polymeric hydrogels are commonly investigated ink materials for 3D and 4D bioprinting applications, as they can contain intrinsic properties relative to those of the native tissue extracellular matrix and can be printed to produce scaffolds of hierarchical organization. The incorporation of nanoscale material additives, such as nanoparticles, to the bulk of inks, has allowed for significant tunability of the mechanical, biological, structural, and physicochemical material properties during and after printing. The modulatory and biological effects of nanoparticles as bioink additives can derive from their shape, size, surface chemistry, concentration, and/or material source, making many configurations of nanoparticle additives of high interest to be thoroughly investigated for the improved design of bioactive tissue engineering constructs. This paper aims to review the incorporation of nanoparticles, as well as other nanoscale additive materials, to printable bioinks for tissue engineering applications, specifically bone, cartilage, dental, and cardiovascular tissues. An overview of the various bioinks and their classifications will be discussed with emphasis on cellular and mechanical material interactions, as well the various bioink formulation methodologies for 3D and 4D bioprinting techniques. The current advances and limitations within the field will be highlighted.

## 1. Introduction

Tissue engineering focuses on the use of multiple cell lines and other biological constituents together with biomaterial-based platforms for the fabrication of artificial organ-like structures, which could restore, improve, or regenerate damaged tissues [1,2]. The biomaterial-based scaffolds should have biocompatible character, controllable degradation rate, low immunogenicity, and mechanical attributes that are equivalent to those met in the respective native tissue. Moreover, cells require a complex three-dimensional (3D) microenvironment to demonstrate their physiological functions. Therefore, the refinement of the fabrication techniques of scaffolds to mimic the architecture of the real tissues and organs is of growing interest [3]. Τhe 3D printing technology is such a technique that has recently attracted great attention. It involves the layer-by-layer deposition of a printable solution (ink) into high-precision and high-accuracy constructs with a specific hierarchy, following a computer-aided design (CAD) schematic, which has been produced either by a graphic design program or by medical diagnostic imaging, such as magnetic resonance imaging (MRI) and computer tomography (CT), depending on the required complexity and precision [4,5,6].

Despite the flexibility of the conventional 3D printing manufacturing techniques, they have some limitations regarding their involvement in biological applications, such as the fact that the 3D printing process does not usually take place in fully sterilized conditions. Thus, postprocessing sterilization is needed, which could affect the mechanical integrity of the scaffolds by altering the chemical bonding status their constituents or leave toxic traces after its conduction, with possibly detrimental effects on cell viability [7,8]. Moreover, the cell seeding process onto 3D-prefabricated scaffolds cannot fully mimic the cellular distribution and homogeneity patterns present in the native tissue, as cells under in vivo conditions are mostly embedded inside complex extracellular matrix formulations [9,10]. This in turn could influence the physiological cellular migration process but also affect the cell differentiation potential within the scaffold, as topology is a crucial parameter in intracellular signaling, growth, and directionality toward various lineages [11,12].

To overcome these shortcomings, a new method of 3D printing has emerged, designated as 3D bioprinting, which is a fabrication process that enables the mixing of living cells with biocompatible materials, usually hydrogels, into a singular construct called bioink. In 3D bioprinting, cells and other biological elements are entrapped inside the printable mixture, replicating, at least partially, the physiological 3D environment that the native tissue exhibits [13,14]. This approach has significantly advanced the tissue engineering research field as it has substantially amplified the efficacy of the produced medical devices, while simultaneously drastically reducing the cost and time of production [15,16]. Bioinks have been prepared so far by utilizing different natural polymers [13,17], by combining natural and synthetic biopolymers [18], or even by employing purely synthetic blends [19], as polysaccharides usually demonstrate a good biological response but lack the necessary mechanical stiffness expected from a biomimicking scaffold. Additionally, based on the physicochemical mechanisms that govern the deposition of the bioink molecules, 3D bioprinting could be divided into four categories: extrusion-based [20,21], inkjet-based [22,23], stereolithography (SLA)-based [24,25], and laser-assisted 3D bioprinting [26,27]. Each of these techniques has advantages in particular areas, such as ensuring minimal cell loss during printing and high printing resolution, while others are more efficient in viscosity issues that stem from the immiscibility or high gelling capacity of the bioink-based ingredients [28].

Because there is an obvious need to continuously develop biomaterials with improved properties, research groups worldwide are constantly working on optimizing the biomechanical attributes of scaffolds by fine-tuning the material compositions or by carefully adjusting various parameters regarding the processing of the materials. An efficient way to enhance both the mechanical and the biological properties of polymeric and nonpolymeric scaffolds can be achieved by the addition of only small amounts of nanobiomaterials into the final product, which after the inclusion of the nanoparticles is referred to as a nanocomposite [29,30,31]. This route presents great versatility in the designing of new biomaterial compositions for 3D bioprinting, in which the integration of nanoparticles can not only amplify the produced bioink biochemical response, but also improve the accuracy, fidelity, and reproducibility of the 3D printing process itself [17,22,26]. However, the presence of nanoparticles can also lead to some adverse effects for the resulting scaffolds, such as reduced biocompatibility [32] and slower degradation rates [33]. This is why the advancement of the 3D nanocomposite bioprinting field aims to refine the production process of biofunctional nanocomposite scaffolds that can elicit the beneficial properties of the integrated nanoparticles, while simultaneously keeping the negative impact of nanoparticles to a minimum [34,35]. There is a wide array of different nanomaterials that have been proven to display biofunctional behavior, depending on their concentration, shape, dimensions, and overall properties [30,36]. In regards to their chemical composition, they can be broadly classified into the following groups: (i) nanoparticles based on carbon, such as graphene and graphene oxide [37], carbon nanotubes [38] and carbon nanofibers [39], (ii) ceramic nanoparticles ranging from silica-based nanobiomaterials [5,40] to calcium phosphate nanoparticles [30,41] as well as various oxides and bioactive glasses [42,43,44], (iii) natural and synthetic biopolymeric nanoparticles [45,46], and (iv) various metallic nanoparticles [47,48,49].

Extrusion-based bioinks may suffer from inconsistencies during the bioprinting process, which can be attributed to the lack of mechanical stiffness of the base constituents or their immiscibility during the preparation phase, thus leading to low-resolution 3D-printed structures [50]. To overcome these limitations, new extrusion bioprinting techniques have emerged, such as the freeform reversible embedding of suspended hydrogels (FRESH). This method facilitates 3D bioprinting of soft and liquid-like bioinks using a sacrificial thermoreversible bath of a soft biomaterial, such as gelatin, as the printing surface. Subsequently, the main bioink is 3D-bioprinted inside this soft matrix, which is able to keep it in place and significantly increase the overall printing fidelity of the extrusion. After the bioink becomes stable, the bath can be removed [51,52]. Another direct extrusion bioprinting approach recently introduced is cryobioprinting, which enhances printing resolution and provides the possibility of the long-term storage of a bioink for use at a later time point. It involves the use of cryoprotectant substances that can preserve cells by halting their metabolism. Additionally, the formation of ice can contribute to the creation of big internal porous cavities, which can improve cell proliferation inside the bioink [53]. Cryobioprinting has been employed to successfully cryobioprint highly precise 3D scaffolds consisting of gelatin methacryloyl (GelMA) and three cell types, C2C12 myoblasts, NIH/3T3 fibroblasts, and human umbilical vein endothelial cells (HUVECs), in order to fabricate muscle–tendon and muscle–microvascular biomimicking units [54]. Although a small portion of the embedded cells were still frozen during the printing process, the rest exhibited excellent proliferation capability, while the spreading angle distribution experiments revealed that the cells had adopted similar elongation and coalignment profiles as those met in the native tissues.

Apart from the mechanical strength that the integration of any nanoparticles can evoke in a polymeric matrix, as a universal feature, some of them are also capable of exhibiting additional properties, when the necessary stimulation conditions are present. For example, there are different 3D-printed nanocomposites that can alter their behavior in the presence of various stimuli, such as temperature [55], pH [56], mechanical stresses [57], and electrical or magnetic fields [58], which is indicative of the impact that nanomaterials can have on a large system, even in small quantities. As such, lately, the term “4D printing” has been established in the literature, which, by definition, refers to how a 3D-printed material can transmute into a construct with additional functionalities, under particular exogenous conditions in their environment [59]. In regards to bioprinting, 4D bioprinting presents an innovative perspective, which aims to enable the production of environmentally responsive bioinks and thus to pave the way for further advancement and optimization of the current artificial organ implants [60].

The principles and significance of the 3D bioprinting technologies, as well as the role of nanobiomaterials in tissue regeneration, have been described in the literature, but often as distinct areas of research interest. This review aims to underline the latest advancements in the fabrication of bioink nanocomposites and their role toward the development of novel personalized medicine devices. To attain this goal, we discuss (i) the establishment of the basic principles of 3D bioprinting techniques, analyzing their advantages and disadvantages, (ii) the main categories of nanoparticles, nanocomposites, and relevant composite bioinks with their contribution in tissue engineering, and (iii) the methods that enable the mixing of bioinks and nanoparticles into biofunctional 3D-printed nanocomposites, designed specifically to accommodate the regeneration of bone, cartilage, dental, and cardiovascular tissues. A discussion will point out the current limitations of the contemporary 3D bioprinting techniques and how the integration of nanocomposites can improve materials properties and effects to overcome such hurdles.

## 2. Materials as Nanocomposites for Printing and Bioprinting

### 2.1. Ceramics

Bioactive nanoceramics is the most thoroughly investigated family of nanocomposites in tissue engineering, which, at lower concentrations, can retain good biocompatibility, direct the maturation of undifferentiated cells toward a specific tissue type, and enhance the mechanical integrity and the degradation profile of the scaffolds into which they are incorporated [61]. One of their most important subcategories are calcium phosphates (CaPs), which display chemical compositions that closely resemble those of mature calcified tissue. More specifically, CaPs are essential for the formation of hydroxyapatite, which is one of the main components of native bone together with collagen; thus, it is usually employed as a biomaterial for bone regeneration [62]. Moreover, one of the three zones that constitute articular cartilage tissue is also calcified; thus, CaP inclusion can be utilized for the restoration of that region [63,64]. So far, nanohydroxyapatite (nanoHAp) has been one of the most common materials for bone and cartilage 3D bioprinting, as it is easily accessible, has a relatively low cost, and presents good dispersion levels in aquatic solutions [27,34,65]. For example, nanohydroxyapatite has been combined with gellan gum and alginate to formulate bioinks that promote chondrogenesis [66]. The integration of nanoHAp amplified the printability of the bioinks compared to the counterparts without nanoHAp by increasing the shear-thinning behavior, which is crucial for extrusion 3D bioprinting as it minimizes cell death during printing. Moreover, the addition of nanoHAp led to higher chondrogenic potential, without compromising the cell viability.

Tricalcium phosphate nanoceramics (TCPs) are another category of calcium phosphate-based biomaterials, with similar bioactivity as that of nanoHAp, which have also found application in 3D bioprinting [67]. Βeta-tricalcium phosphate (beta-TCP) has been incorporated into GelMA and alginate bioprinting solutions together with bone marrow mesenchymal stem cells (BM-MSCs) to evaluate the role of nanoparticles in chondrogenesis and how they can alter the printing conditions [35]. Scaffolds that had beta-ΤCP nanoparticles showed upregulation of various chondrogenesis-related factors, such as the proteoglycan aggrecan and different types of collagen proteins col-1, col-2, and col-10 that are present in native cartilage tissue, while also demonstrating greater 3D printing shape fidelity.

Bioactive glasses (BGs) are amorphous silica-based bioactive nanomaterials, with osteoinductive and osteoconductive capabilities, which can evoke bone-like apatite formation and upregulate bone tissue-related markers [68]. In their basic form, they consist of a SiO_2_–CaO–Na_2_O–P_2_O_5_ chemical sequence, containing all the necessary basic ingredients for the construction of hydroxyapatite. Additionally, depending on their fabrication method, BGs can have varying effects on their biological response [69]. Due to their tailorable biomechanical properties, BGs have attracted a lot of attention in tissue engineering, and especially in the 3D bioprinting field [70,71]. Bioactive borate glass has been combined with poly(lactic acid) (PLA), and the mixture was printed in parallel with an alginate/gelatin bioink containing adipose-derived stem cells (ADSCs) to assess the construct’s cytocompatibility [72]. Although the BG/PLA 3D-printed compartment did not include any cells, the idea behind this work stemmed from the hypothesis that the degradation of PLA would allow for the gradual release of the bioactive glass from the PLA 3D-printed-like constructs, which could come into contact with the adjacent cell-containing bioink and subsequently affect its biological response. Indeed, the presence of the BG nanoparticles in the PLA 3D-printed scaffolds not only provided increased mechanical support to the complex multicomponent system but also positively influenced cell proliferation inside the bioink. Based on this methodology, other groups have also prepared similar multicomponent systems by sequential 3D bioprinting/3D printing layer deposition with similar results as the abovementioned work [73,74].

Laponite is another silica-based nanomaterial with a basic chemical formula of Na^+0.7^[(Si_8_Mg_5.5_Li_0.3_)O_20_(OH)_4_]^−0.7^ that possesses multiple biofunctional properties. Due to sodium release in aquatic solutions, laponite adopts a negative charge, which can interact with many biological constituents impacting their physiological behavior [75,76]. Moreover, when laponite degrades, its different ions are released, including magnesium ions, silicic acid, and lithium ions, which have been found to affect both osteogenesis [77] and angiogenesis [78] pathways, making it a promising material for 3D bioprinting technologies [40,79]. As bone is a vascularized tissue, it is crucial for any potential medical device that is aimed at bone regeneration to simultaneously elicit both bone formation and vascularization in the presence of undifferentiated cells. In this respect, laponite was mixed with GelMA biopolymer and BM-MSCs into a final 3D bioprintable solution, which was designed for vascularized bone restoration applications [80]. After photopolymerization inside the range of the visible light spectrum, the final bioinks were formulated and evaluated biomechanically. Although the incorporation of laponite did not induce any shear-thinning properties in the bioink, it substantially increased the printing fidelity and mechanical robustness of the scaffolds. Additionally, the laponite-containing bioinks depicted significantly enhanced cell viability compared to the photopolymerized GelMA control samples. Regarding the bioinks’ angiogenic potential evaluation, an ex vivo implantation of the supplemented with VEGF implants was conducted into chicken chorioallantoic membrane without disrupting it, with the results indicating that the laponite-containing bioinks achieved a densely penetrating vasculature network that protruded out of the scaffolds, and this was more prominent compared to the GelMA bioinks. 

### 2.2. Carbon-Based Nanoparticles

Graphene is a nanomaterial that consists of carbon atoms, which form a 2-dimensional sheet that resembles a honeycomb-like arrangement [81]. Due to its innovative structure, it has revolutionized material research tremendously, with multiple applicability ranging from renewable energy technologies and energy storage devices to microelectronics and biotechnology [82]. Graphene also displays spectacular electrical conductivity, large surface area, and mechanical strength, three distinguishable characteristics that make it a potent candidate for the total replacement of silicon, on which almost all contemporary electronic devices are based [83]. For further utilization, graphene is often oxidized to form graphene oxides (GOs), with ratios of graphene toward oxygen molecules greater than two [84]. Although pure graphene is only slightly biocompatible, its oxide formations have been found to depict acceptable cytocompatibility levels at low concentrations, with the constructs into which it is incorporated still retaining all its useful attributes [85,86]. Due to its appealing nature, graphene has been investigated lately as a prospect for 3D bioprinting applications. Its excellent electroconductivity is believed to affect the lineage directionality of undifferentiated cells, while its mechanical properties can contribute to the optimization of the extrusion 3D bioprinting process [87,88,89]. A polyurethane (PU) elastic polymer was prepared by three different synthetic copolymers, poly(ε-caprolactone) diol, poly(D,L-lactide) diol, and isophorone diisocyanate, which was mixed with graphene in two forms, either as GO or as graphene platelets combined with pluronic, with the latter being introduced as an alternative method of increasing graphene molecule dispersity in water [90]. Then, the three solutions were mixed with cellular suspensions of neural stem cells (NSCs) and were subsequently 3D-bioprinted. Surprisingly, the presence of graphene led to a drop in the storage modulus (G′) and loss modulus (G″) values, nevertheless bringing them closer to the elastic nature of the physiological neural tissue. The cytocompatibility level of the graphene-containing bioinks was significantly higher compared to that of the PU control, while the various neuronal differentiation markers examined showcased higher upregulation in the case of two graphene bioinks, with the graphene/pluronic composition retaining the best biological response out of the two. These results were also validated by a series of immunochemistry assays.

Carbon nanotubes (CNTs) and carbon nanofibers (CNFs) also share similar properties as those of graphene, with the CNTs being cylindrical folded sheets of graphene monolayers and CNFs demonstrating a less canonical and more erratic stacking pattern compared to CNTs [91]. However, their shape and architecture make them useful in a wide range of tissue engineering applications, which sometimes can be more favorable compared to the integration of pure graphene, as geometrical properties also play a role in the successful functionalization of medical devices [38,39,92,93,94]. By employing a hybrid 3D printing/3D bioprinting technique, a collagen-methacrylated and human coronary artery endothelial cell-consisting bioink was created, which was subsequently co-3D-printed with another solution containing a mixture of alginate and CNTs but without cells. This aimed to produce a multilayer composite, with alternating sequences of 3D-bioprinted and 3D-printed lines, in order to avoid direct contact of the CNTs with the cells due to cytotoxicity issues but still elicit a mechanical and electrical enhancement of the final scaffolds [13]. The compression modulus of the scaffolds is enhanced by increasing the electroconductivity, and the presence of CNTs had a positive effect on both cell proliferation and differentiation of the cells, thus presenting an innovative patch for cardiac tissue restoration.

### 2.3. Polymeric Nanoparticles

Most of the natural and synthetic biopolymers are used for the preparation of macroscopic scaffolds and not as nanoparticles. An exception is nanocellulose, a biocompatible polysaccharide whose chemical structure consists of repeating β (1→4)-linked D-glucose units derived from different biodegradable sources, able to form either nanoscale crystals (CNCs) or fibrils (CNFs). Apart from its low cytotoxicity, nanocellulose depicts thixotropic behavior under external mechanical probing and displays a high gelling capacity at room temperature [95]. Due to these attributes, its incorporation into various hydrogels, either in its pure form [96] or after chemical modifications [97], has been adopted with great interest by the tissue engineering community. The role of CNCs and CNFs in 3D-bioprinted constructs, especially for the regeneration of cartilage, has been extensively investigated in the literature [98,99,100,101]. In one such work, CNFs were mixed with alginate at four ratios, and the resulting solutions were allowed to homogenize with cell suspensions of human nasoseptal chondrocytes (hNCs) in order to facilitate the preparation of different bioprinting solutions [102]. After the 3D bioprinting extrusion, the bioinks were formed by immersing them in a CaCl_2_ solution. As expected, the presence of CNFs amplified all rheological properties of the resulting bioinks, while the cell proliferation was increased over time. In another work, CNCs were encapsulated inside chitosan, and the mixture was blended with MC3T3-E1 pre-osteoblasts to formulate bioinks with potential osteogenic capabilities [103]. Once again, the integration of CNCs inside chitosan greatly increased the stability and printability of the bioinks, compared to the pure chitosan counterparts. For their osteogenic evaluation, alkaline phosphatase activity, collagen production, and calcium mineralization levels were determined, revealing maturation of the cells toward osteoblasts.

Poly(3,4-ethylene dioxythiophene) (PEDOT) is a nanosized synthetic polymer, with an excellent biocompatibility profile [104]. PEDOT is electroconductive and piezoelectric but highly hydrophobic; therefore, other hydrophilic substances, such as poly(styrene sulfonate) (PSS), are needed to make it more water-soluble [105]. Due to its high electrical conductivity, PEDOT is often employed in tissue regeneration applications that require the flow of bioelectricity, such as neural [106,107], myogenic [108], and cardiac tissues [109]. In an interesting work, which aimed at the restoration of spinal cord injuries, PEDOT with sulfonated lignin (PEDOT:LS) was mixed with GelMA, hyaluronic acid methacrylate (HAMA), and neural stem cells (NSCs), with the bioinks forming after photopolymerization [110]. In vitro evaluation of the scaffolds revealed that the PEDOT:LS bioinks promoted neuronal differentiation in a much more efficacious manner compared to the nonconducting scaffolds. These results were in line with the in vivo findings, demonstrating that after 12 weeks from the insertion of the 3D-bioprinted constructs into the spinal cords of mice, the electroconductive nature of PEDOT contributed to the formation of long nerve fibers leading to a partial regeneration of motor function of the mice hindlimbs. Other 3D bioprinting works revolving around the use of PEDOT have also illustrated its advantages in regenerative medicine research [111,112].

### 2.4. Metallic Nanoparticles

**Figure 1:**
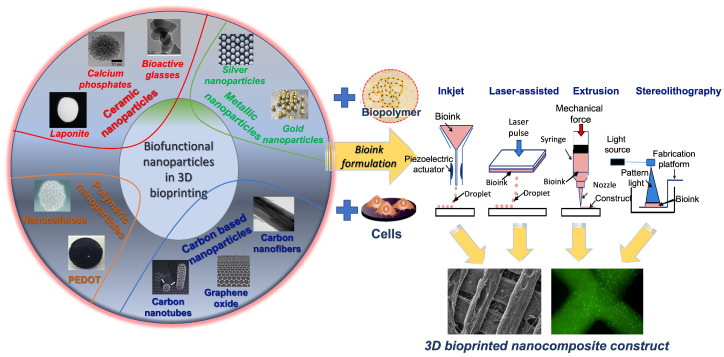
Schematic showing the different types of nanomaterials (ceramic-, metallic-, polymeric-, and carbon-based) that can be combined with biopolymers and cells resulting in 3D-bioprinted nanocomposite constructs by means of the printing methods including inkjet, laser-assisted, extrusion-based, and stereolithography.

Among the different available metals, some have been identified to possess biological properties [113]. One such metal is gold, whose nanoparticles (AuNPs) have good biocompatibility, significant electrical conductivity [114], and antibacterial character [115]. For this reason, gold nanoparticles have been used for the designing of cytocompatible platforms with different utilities, ranging from the construction of tissue engineering scaffolds with tunable electrical conductivity for bone [116], neural [117] and cardiac regeneration [118] to an ingredient for bioelectronic devices [119,120]. Silver nanoparticles (AgNPs) also exhibit similar traits as those found in AuNPs, which make them suitable for equivalent applications [121,122,123,124]. One serious drawback of most of these metals is their lack of biodegradation, at least in their pure form, which significantly limits their use for in vivo applications [33]. This explains the fact that the literature concerning the implementation of metallic nanoparticles for 3D bioprinting applications is quite scarce. In one such work, a comparative study was conducted with AuNPs and metal carbide nanosheets, in order to deduce their behavior when entrapped in a bioink consisting of GelMA and skeletal muscle cells C2C12 [125]. Both nanocomposites demonstrated an augmented response in regards to both conductivity and printing fidelity compared to the pure GelMA bioink. Moreover, these electrical properties were found to depict an increased effect on the differentiation of the embedded myoblasts. Moreover, another report described strontium-doped gelatin nanocomposite bioinks for bone regeneration, by preparing four different concentrations of self-assembly-based nanoparticles and integrating them in bioinks consisting of GelMA and human mesenchymal stromal cells (hMSCs) [126]. The presence of strontium revealed a substantial improvement of the mechanical properties compared to the control GelMA bioink, with the increase in viscosity resulting in higher stability and precision fidelity during the extrusion process. Additionally, the various strontium concentrations, especially the 1.5 mg/mL, showed a significant upregulation of osteogenesis-related markers, such as alkaline phosphatase activity, Runx-2, and osteonectin. Figure 1 shows a schematic of the different types of nanomaterials (ceramic-, metallic-, polymeric-, and carbon-based) used for composite bioprinting.

## 3. Three-Dimensional Bioprinting Techniques

In the following, the four main bioprinting techniques will be described. 

### 3.1. Extrusion-Based 3D Bioprinting

Extrusion 3D bioprinting is the most common technique for 3D bioprinting, which enables the mixing of cells with various biopolymers and the subsequent 3D printing of these bioinks into architecturally intricate biomimicking constructs with tunable mechanical properties [127]. The prepared bioink is usually stored in a syringe, and following a CAD, it is deposited layer-by-layer through nozzle tips of different diameters. All extrusion-based bioprinters have integrated systems of controlled pressure and temperature in order to accommodate the printing parameters that are required for each distinct composition [128]. This technique is suitable for the production of medical devices in large scale, as the ejection of the bioink takes place continuously, reinforcing the mechanical stiffness of the scaffolds. Extrusion 3D bioprinting also excels at bypassing viscosity issues, by increasing the pressure or the temperature during the printing process. However, the gradual buildup of extrusion pressure inside the walls of the dispensing nozzle may deteriorate the printing fidelity and the cell viability of the printable bioink [129,130]. The increased viscosity in nanocomposite bioinks can affect printability with different techniques as well as cell viability with extrusion-based 3D bioprinting due to increased shear stresses [131]. 

### 3.2. Inkjet 3D Bioprinting

Inkjet 3D bioprinting. Inkjet 3D bioprinting is another bioprinting method based on a drop-on-demand ejection system of bioink droplets, by utilizing different forces, such as piezoelectric, thermal, and electromagnetic, to propel the bioink into well-defined 3D shapes. Because it does not require direct contact with the deposition surface in order to function, this technique favors the interweaving of different biological constituents, by enabling the parallel 3D bioprinting of heterogeneous solutions, such as drug and gene carriers containing bioinks [132,133]. This in turn facilitates faster printing with higher accuracy and a negligible impact on the encapsulated cell viability [134]. Conversely, inkjet bioprinting is weighed down by the need for solutions of low viscosity, making its applications rather impractical for bioinks with a high gelling capacity [28]. 

### 3.3. Stereolithography 3D Bioprinting

Stereolithography-based bioprinting relies on an older traditional method of printing that uses a photopolymerizable solution as a precursor in liquid form, which is poured over a mold with the desired architecture. Subsequently, as the liquid solution adopts the shape of the predesigned solid volume, it is then irradiated by laser or UV light in order to initiate the photocrosslinking process and force the solidification of the bioink. Following a CAD, the stereolithography-based bioprinter keeps repeating these two steps until the final structure is solidified and resembles a hydrogel-like structure [135]. Although this bioprinting method provides great printing resolution, it is rather slow due to the alternating cycles of bioink injection and irradiation curing phases. Additionally, cell viability can also be compromised because laser and UV irradiation energy can potentially damage various cellular components or disrupt their physiological processes [136,137].

### 3.4. Laser-Assisted 3D Bioprinting

Laser-assisted bioprinting is quite similar to inkjet bioprinting in principle, as it does not require direct contact with the deposition surface. A laser beam is directed through several different angled mirrors to the bioink droplet, heating it and therefore facilitating its mobilization toward a surface target. Following a layer-by-layer approach, the aforementioned process is repeated multiple times, until the stacking of cohesive layers formed by adjacent droplets takes place, constituting the final bioprinted scaffold [138]. In return, this technique enables high-velocity printing and precision, and can substantially upscale the upper limit in the population of cells that can be incorporated in the bioink, making it suitable for applications that require high initial cell numbers [139,140]. However, the laser beam source can negatively affect the initial cell viability due to the heat absorbance from the droplet [141]. An overview of the advantages and disadvantages of the bioprinting techniques is summarized in Table 1. 

## 4. Nanocomposite Bioprinting Applications

### 4.1. Conventional Bioink Role and Properties for Bone and Cartilage Composite Bioprinting

Various combinations of bioactive, natural, and synthetic materials have been investigated to 3D-print multifaceted nanocomposites as implantable therapeutic constructs for bone and cartilage tissue engineering. These nanocomposites aim to improve the physical stability and biological functionality of the scaffold during both the fabrication and application steps. For the 3D printing of bone replacements, of importance is to develop a scaffold that can provide high mechanical properties relevant to that of native bone while supporting osteogenic cell differentiation and bone mineral deposition for regenerative purposes. Another major focus in the field is to tune the degradation rate of the implanted scaffold to align with the regeneration rate of newly formed bone post injury. To do so requires a careful balance of complex interdisciplinary material properties, such as crosslinking density, release kinetics, and physicochemical parameters. For the former consideration, synthetic thermoplastic polymer bases for nanocomposite inks have been widely researched for their biocompatibility, biodegradability, and superior mechanical strength. Such commonly researched polymers in bone tissue regeneration include PCL, poly(lactic-coglycolic acid) (PLGA), poly(ethylene glycol) (PEG), and PLA [142]. While these materials provide excellent mechanical properties necessary for adequate bone repair and regeneration, they are limited in their ability to incorporate pertinent biological cues, such as cells, growth factors, and vascular permeability for active remodeling. Additionally, such materials can face interruptions and structural jeopardy during various 3D printing methodologies. Alternatively, soft hydrogels generally possess lower mechanical properties but intrinsic hydrophilic properties, ECM-like biomimicry suitable for clinical applications, tunable biodegradability, and higher potential for increased biocompatibility. Specifically, 3D bioprinting focuses on the development of a bioink encapsulating growth factors, live cells, and biomaterials to 3D-print precise native tissue-like scaffolds that pose high potential for active repair once implanted. Careful consideration of printing parameters must be observed to retain cell viability during and after printing, including temperature, shear and thinning forces, cytotoxicity, and material compatibility. For this reason, biocompatible hydrogels capable of housing live cells are frequently employed as bioink bases and are enhanced with additional biological materials [143,144] in the form of nanoparticles. Examples of commonly used naturally derived polymers include alginate, gelatin [145], hyaluronan, gellan gum, and collagen. These polymers and others alike are advantageous in their ability to retain water and exhibit smooth shear-thinning properties that aid in printing fidelity. To address the inherent lack of mechanical properties in these hydrogels and others similar, the synthesis of such polymers has been explored with a variety of inorganic and organic nanoparticle additives, aimed to tune the structure and biological activity of the bulk printing material. Common inorganic particle additives have included hydroxyapatite, bioactive glass, silicates, nanoclays, and graphene oxide, which have been utilized to improve the printability of inks and shape fidelity. The combination of these different hydrogels with composite nanomaterials to develop 3D-printed bioinks and their associated printing, mechanical, and cellular compatibility properties will be discussed below, specifically for application in bone tissue engineering and repair. 

### 4.2. Nanomaterial Composites for Bone Bioprinting

One of the main interests in the field is to explore the potential of particle additives in increasing the yield strength properties of the selected base hydrogel. In one example, the incorporation and functionality of mesoporous silicate SiO_2_–CaO nanoparticles were explored with an alginate–gelatin hydrogel blend for the 3D printing of bone tissue repair scaffolds [146]. Notably, constructs demonstrated prominent surface hydroxyapatite formation suggesting positive potential for osteoconductivity, while stimulating osteoblast proliferation and differentiation in vitro. Although successfully multifunctional in terms of bioactivity, the mechanical properties of the hydrogel-based nanocomposite constructs still showed room for mechanical improvement. This nanocomposite system was also able to be loaded with a therapeutic osteogenic drug that was released in vitro, demonstrating the versatility and wide clinical potential of such systems. A similar approach investigated the incorporation of bioactive glass particles into methacrylated collagen for printing live mesenchymal stem cells as a bioink for bone repair constructs [147]. As collagen has available cell adhesion sites and presents high cytocompatibility, porous constructs were extrusion-printed with high cell viability with the aid of a thermoreversible support bath. The incorporation of bioglass particles was found to improve the rheological properties of the material for improved printing and osteogenic differentiation of MSCs in vivo but did not contribute to a significant enhancement in the material mechanical properties possibly due to limitations in photocrosslinking. Another group demonstrated similar findings with the use of methacrylated alginate and human allograft bone particles 3D-printed with human mesenchymal stem cells [148]. Notably, compared to the prior study, the use of allograft bone particles was found to contribute to enhanced cell viability during and after printing, for 90% viability for up to 28 days in vitro. Moreover, the potential for graphene oxide additives to improve bioink mechanical properties was explored in several applications with alginate- [149], gelatin- [150], alginate/gelatin blend-, and gellan gum-based hydrogels [151]. Generally, the addition of graphene oxide was found to contribute mechanical strength and improve the bioink printing performance, and in several applications, was tuned in accordance with the hydrogel with chemical modifications to function in targeted drug delivery and tumor suppression.

Silica-derived nanoparticles are widely employed as nanoparticle additives due to their osteogenic activity and potential to modulate ink printing properties. Additionally, silica nanoparticles can be covalently bound to their base polymer, which allows for further tunability of rheological properties and improving printing fidelity [152]. In one application, photocrosslinkable gelatin methacrylate was integrated with mineral-enhanced mesoporous nanosilica spheres (MSN) [153] as a bioink for extrusion 3D printing. Specifically, the silica nanoparticles were functionalized with calcium, phosphate, and dexamethasone for their additional pro-osteogenic properties. The bioink was successfully printed with human bone marrow-derived mesenchymal stem cells, where in vitro studies uncovered an efficient printability window supportive of osteogenic stem cell differentiation. Another group explored adjacent osteogenic potential through an integrated nanocomposite ink, consisting of functionalized copper-doped mesoporous bioactive glass nanoparticles and an alginate–gelatin base [154]. The cytocompatibility and directed differentiation capacity of the construct was evaluated by loading the ink with several types of osteoblasts, including bone marrow stromal stem cells and human umbilical vein endothelial cells. In vitro studies showed that the nanocomposite ink supported osteogenic differentiation and angiogenesis. Additionally, the constructs demonstrated favorable rheological properties, enhanced shape retainment, and consistent stability during extrusion printing. A similar approach incorporated positively and negatively charged self-assembling nanosilicates within a gelatin–alginate hydrogel [155]. The addition of laponite-derived nanosilicates contributed to improved printability due to the shear-thinning material properties and overall mechanical strength of the construct by increasing the compressive modulus. Importantly, the bioink was found to support the osteogenic differentiation of human mesenchymal stem cells for up to two weeks in a rat defect model. This demonstrated an efficient approach to overcoming one of the major challenges in utilizing hydrogel bioink materials, which is the acquisition of sufficient mechanical properties while maintaining cell viability.

### 4.3. Nanomaterial Composites for Cartilage Bioprinting

As cartilage is a hypocellular tissue with extremely limited regeneration potential, several 3D bioprinting approaches with a variety of nanocomposite additives have been researched for their chondrogenic and therapeutic potentials. One of these examples includes the integration of methacrylated gelatin layered with layered hydroxide nanoparticles for the formulation of highly printable hydrogel bioink [156]. Here, hydroxide particles were investigated for their mechanical properties that may be able to assist the soft nature of methacrylated gelatin to provide a mechanically durable printed construct. Using extrusion 3D printing, it was found that the inclusion of hydroxide particles improved the mechanical properties of printed scaffolds by almost 50%, while supporting osteoblast survival and proliferation. The layered hydroxide nanoparticles also enhanced cell proliferation, which needed to be tuned in accordance with particle concentration to not induce aggregation of materials during printing. A particle concentration of 3% *w/v* was found to produce good printing stability with adequate rheological properties. With such mechanical properties that can be further tuned with particle concentration, this bioink could serve as a substitute for cartilage and/or bone tissue. Other bioprinting approaches have investigated the printability of active inks with various stem cells, including adipose-derived MSCs. Inducing the sustained chondrogenic expression of MSCs post printing and hypothetically upon clinical application remains a challenge. One way the differentiation of MSCs has been attempted to be more precisely directed was with the formulation of a nanocomposite hydrogel containing piezoelectric barium titanate nanoparticles and graphene oxide nanoflakes [157]. These materials were investigated as they could be stimulated with controlled ultrasound waves at a variety of frequencies and intensities to direct the chondrogenic differentiation of ASCs. A range of adequate stimulation was characterized in accordance with ASC viability, in which a reduction in inflammatory cytokines and chondrogenic ASC differentiation over a period of 10 days was observed. Further characterization of such an approach that utilizes external stimuli of materials to induce targeted cellular differentiation aligns with many goals of 4D bioprinting and could serve as inspiration for future cartilage bioprinting approaches. Other approaches for cartilage bioprinting have incorporated materials discussed for bone bioprinting, such as gelatin/alginate hydrogel blends incorporated with zinc/silica nanoparticles [158], gelatin/sodium/alginate blends with nanohydroxyapatite particles [159], and gellan gum/alginate incorporated with silk nanoparticles [160]. Indeed, much overlap exists between cartilage and bone bioprinting research, which allows for the insightful crosstalk of ideas and approaches that can inspire new ideas within each field.

With these diverse approaches, a trend is seen in which the nanocomposite additives contribute to stability and improved printing fidelity of the hydrogel base along with some improvement to the material mechanical properties. Because the mechanical properties of bone are high relative to that of softer tissues in the body, it remains a challenge to achieve these levels with soft hydrogels, although exciting progress is being made with the exploration of various nanoparticle materials. Another consideration for osteochondral implantable scaffolds should be the spatial microarchitecture of the printed construct, such as the distance between cells and scaffold gap junctions that affect paracrine signaling, and thus the chondrogenic and/or osteogenic capacity of such constructs upon application [161]. Specifically, the characteristics of the transition region from bone to cartilage and its associated structural and mechanical properties that may exist in a gradient manner, should be taken into consideration for osteochondral implants. Recapitulation of these heterogeneous microarchitectures can be investigated with varying material concentrations and, therefore, properties at predetermined locations of a printed structure. Additionally, the microarchitecture can be designed to be conducive for vascularization, an essential formation for successful construct integration to native tissues [162]. Future research may explore the combination of nano- and microparticle additives with consideration to the physical interactions between material and live cells, in addition to other material modifications that may further enhance the overall load-bearing properties. Exploring these characteristics with in vivo studies will help further comprehend the potential of such composite bioinks. 

### 4.4. Three-Dimensional Printing of Bone and Cartilage Constructs with Extracellular Matrix Composites

A natural material of relatively recent interest in osteogenic tissue engineering is the decellularized extracellular matrix (dECM) derived from native tissue and organs. This material has gained high interest due to its superiority in guiding cellular behavior and mimicking niche microenvironments via retained ECM proteins and growth factors [163]. dECM can be harvested from several types of tissues in accordance with the application. It is ideal to match the mechanical and biological properties between the dECM from the tissue source and that of the tissue of application interest, in order to increase compatibility and support cell-specific proliferation. One of the main challenges in utilizing dECM materials is achieving sufficient printability and mechanical and structural properties. Thus, in bone and cartilage 3D printing applications, dECM materials have been utilized in a variety of manners, such as coating to printed nanocomposite scaffolds [164,165] or as a bioink composite base [166]. An example of the latter includes a porcine-derived demineralized bone dECM–PCL composite extrusion-printed for bone regeneration [167]. This ink was advantageous as it harnessed high mechanical properties from synthetic PCL combined with biological instructions from dECM. Porosity and fluid retention was maintained with beta-tricalcium phosphate additive, which contributed to the overall integrative and regenerative capacity of the scaffold. Additionally, the scaffold showed a 20% increase in compressive modulus relative to the PCL control groups, successfully mimicking physical properties of human cancellous bone conducive to osteogenic differentiation. Another group researched the integration of methacrylated porcine bone-derived dECM with alginate to develop a bioactive ink for printing with adipose-derived stem cells [168]. Here, the methacrylation of the dECM material was researched in its ability to increase the printability of ECM, which is one of the current limitations in direct printing of dECM materials. While composite bioinks here showed increased cellular proliferation and osteogenesis, it was evident that the methacrylate concentration needed to be balanced with material viscosity so as to not induce high cell death during printing. Another approach to improve the printability of dECM included combining tendon dECM with synthetic poly(ethylene glycol) diacrylate (PEGDA) [169]. Here, the hybridization of these two materials was investigated for enhanced mechanical properties, printability, and support of MSC viability and proliferation. While this approach did not include a particle-based additive, the integration of synthetic material with natural ECM demonstrated an effective approach to overcome some of the limitations of ECM bioprinting, which can inspire similar approaches with micro- and nanoparticles. In another investigation, porcine small intestinal submucosa (SIS) dECM was incorporated in a Sr^2+^/Fe^3+^-HA printing slurry for cryogenic 3D printing of bone repair scaffolds. SIS dECM has been examined in a variety of bone construct research due to its retainment of collagen, fibronectin, and glyco-proteins and -aminoglycans [170], which produces excellent bioactivity and compatibility. Thus, the printability of the material has attracted recent interest. As a slurry additive, it contributed to enhanced osteogenesis and biomineralization, where the cryogenic 3D printing methods allowed for improved porous microstructure.

As a material coating to a printed structure, dECM can still deliver pertinent niche biological cues without the need to contribute load-bearing functions. A recent example includes a coating of a hydroxyapatite and poly (L-lactic acid) (HA/PLLA) scaffold with porcine bone-derived dECM [165]. The mechanical properties of this biocomposite scaffold were successfully heightened via a series of plasma treatments and thermal annealing and tuned with HA particle diameters. HA particles with diameters on the nanoscale were found to increase the flexural strength of the printed composite by up to 30% relative to HA particles with larger diameters on the micron scale. Increasing the hydrophilicity of the scaffold through in situ plasma treatments during 3D printing allowed for an efficient coating of porcine bone tissue-derived dECM and contributed a 1.5 fold increase in compressive moduli. The scaffolds here were not printed with live cells as thermal annealing and plasma treatments processes during the printing process were not conducive to cell survival. However, cells seeded onto the constructs after printing showed high viability (90%) and osteogenic differentiation for up to 7 days. Osteoinductive microenvironments were also found to be supported by a printed PCL–calcium silicate (CS) scaffold coated with MG63 cell-derived dECM [164]. Surface hydrophilicity, increased by CS, also contributed to efficient dECM coating, supporting adhesion, proliferation, and osteogenic cell behavior. Such recent experiments support the continued research and applications of dECM in bone and cartilage tissue engineering. The success of dECM as an osteoinductive material coating encourages its use in the development of new bioinks, such as further investigation of 3D printing dECM with live cells and the adaptation of dECM into different configurations as nanoscale additive ink materials. These avenues are yet to be fully explored but are becoming more informed by such presented experiments.

In addition to dECM materials, some components or molecules of ECM are utilized as the base components for the formulation of ECM-like composite hydrogels for bioinks. These materials can be commercially obtained and do not need to undergo intensive digestion or decellularization processes as the dECM materials described previously. One example of such an application includes the integration of synthetic peptides of alpha helix amino acids with amorphous magnesium phosphate (AMP) particles [171]. Here, the synthetic peptides were selected for their ability to form a nanofiber structure resembling that of the native extracellular matrix, and AMP particles were selected for their osteoinductive and anti-inflammatory properties. Dental pulp stem cells were incorporated into the ink for microvalve bioprinting of gridded structures, where the addition of AMP particles increased the viscoelastic behavior of the ink during printing and increased the storage modulus by over 50%. Notably, the bioink showed high cell viability up to 90% for five days, and mineralization and osteogenic gene expression without the addition of regularly incorporated chemical inducers were observed over a period of 21 days. For cartilage bioprinting, recent progress has been made in developing ECM-inspired bioinks or bioinks that utilize dECM. One approach investigated the use of a microporous photocrosslinkable methacrylated acellular cartilage matrix modified with gelatin methacrylate, poly(ethylene oxide) (PEO), and PCL [172]. A methacrylated acellular cartilage matrix was derived from pig models and prepared with decellularization and methacrylation methods. PEO here was utilized as a porogen, and PCL was incorporated for supplementing mechanical properties. Multinozzle bioprinting was used to print inks with chondrocytes, where the distribution and viability of printed cells and material were precisely controlled by tuning the extrusion pressure, rate, and temperature of the bioink as to maintain shear-thinning behavior at low temperatures. The parameters were optimized to result in a printed structure with high shape fidelity. Additionally, high cell metabolism and proliferation were maintained during and post printing for up to 90% cell viability for a period of five days post printing. Impressively, quantitative and histological analyses showed that chondrocyte-related markers gradually increased with time in mice models supporting the regeneration of mature cartilage. This approach shows considerable promise for these materials to support clinical chondrogenesis and cartilage regeneration with further exploration.

**Figure 2:**
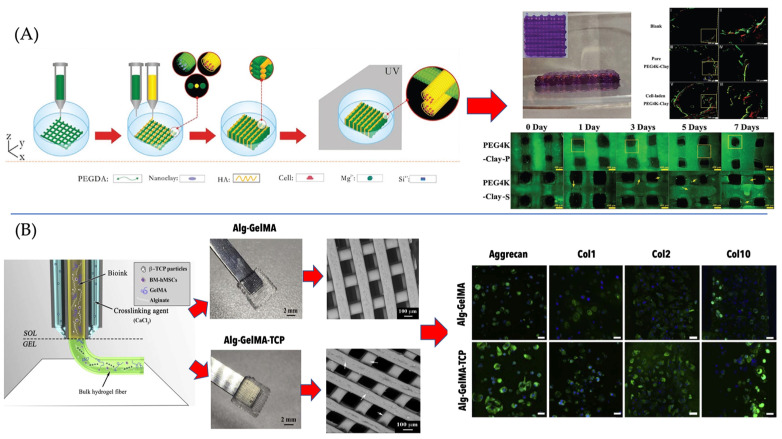
(**A**) Schematic depicts the fabrication technique and the biological response of bioinks comprising poly(ethylene glycol) diacrylate (PEGDA), hyaluronic acid (HA), and laponite (nanoclay) designed for bone tissue engineering; (**B**) displays the preparation of 3D-bioprinted scaffolds containing alginate, GelMA, and beta-TCP for cartilage regeneration (adapted with permission from [35,180], 2022, IOP Publishing, Wiley VCH Verlag GmbH & Co KGAA).

In addition to bone tissue engineering, dECM- and ECM-inspired materials have been utilized as bioink materials for 3D bioprinting for other tissue applications. These have included cartilage, head, and neck tumor modeling [168], skeletal muscle regeneration [173], and liver applications [174,175,176]. One of the limitations in using dECM as nanobased additives to bioinks remains in the ability of the material to maintain shape and biological cues at the nanoscale during the extensive preparation and fabrication processes required. The properties and characterization of dECM informed by experiments that utilize dECM in a variety of forms, such as those described above, provide avenues to overcome such limitations. This encourages the continued investigation of dECM as a nanoparticle and other configurations of additive manufacturing for bioink development and to continue maximizing the potential of dECM’s superior biological properties. The wide investigation for applications and enhancement of ECM materials demonstrates high promise in this material’s ability to reach clinically therapeutic efficacy for treatment of vast human tissue diseases. Table 2 shows various nanocomposite bioink compositions for bioprinting scaffolds and constructs, the cell types used in specific applications, and the material enhancements. Figure 2 presents two strategies for bone (Figure 2A) and cartilage (Figure 2B) nanocomposite bioprinting.

**Table 2:** Representative nanoparticle composite inks and associated enhancements on 3D-printed scaffolds for tissue engineering applications.

Nanoparticle Additive	Bioink Composition	Cell Type	Application	Material Enhancements	References
Silica nanoparticles Calcium-functionalized Copper-functionalized Positively/negatively charged	Gelatin methacrylate Gelatin–alginate Collagen methacrylate	Bone marrow mesenchymal stem cells Human umbilical endothelial cells	Bone	Enhanced alkaline phosphatase cell activity, calcium deposition, and yield stress value.	[147,148,153,155]
Layered hydroxide nanoparticles	Gelatin methacrylate	N/A	Cartilage	Enhanced compressive strength, cell spreading, and cell aspect ratio.	[156]
Piezoelectric barium titanate nanoparticles + graphene oxide nanoflakes	Bioinstructive matrix (RGD-VitroGel)	Adipose-derived mesenchymal stem cells	Cartilage	Enhanced antibacterial effects and chondrogenic cell activity.	[157]
Calcium-modified silicate nanoparticles	Gelatin–alginate- Poly(ϵ-caprolactone)	Wharton’s jelly mesenchymal stem cells Human umbilical vein endothelial cells	Dental tissue	Increased Young’s modulus, angiogenic biomarkers, and bone formation proteins.	[177]
Zirconia nanoparticles	Acrylate ester-based dental resin	N/A	Dental tissue	Increased ductility and flexural strength and enhanced sorption in vitro.	[178]
Silica nanoparticles	Polymer-infiltrated ceramic network	N/A	Dental tissue	Increased shear bond strength and surface free energy.	[179]
Carbon nanotubes	UV collagen methacrylate and alginate	Human coronary artery endothelial cells	Cardiovascular tissue	Enhanced mechanical and electrical properties, increased stiffness, and cell attachment.	[13]

### 4.5. Nanocomposites for 3D Printing and Bioprinting in Dental Tissue Engineering Applications

Dental bone graft materials have been developed over the years to regenerate artificial dental tissues by controlling the augmentation of osteogenesis after tooth loss and guide new bone formation. A successful dental grafting scaffold should be biocompatible and porous with an interconnected network of pores and channels to provide nutrients and oxygen to efficiently support cell growth. The mandible is a functional organ that supports the facial structures and enables mastication and speaking. Large mandible defects may require composite tissue reconstruction, such as the osteocutaneous vascularized free flap, which have the drawbacks of additional surgery and morbidity at the donor site [181]. The recently developed 3D bioprinting technology helps to overcome these limitations using cells, bioactive molecules, and materials with high mechanical strength, resilience, and biocompatibility. Three-dimensional bioprinting appears to be an outstanding manufacturing route to revolutionize personalized medicine.

Ceramic nanocomposites, such as calcium phosphate-based biomaterials, bioactive glasses, and zirconia, have been successfully used as bone and dental replacement materials for over two decades due to their excellent biocompatibility. PCL is a highly biocompatible and biodegradable biomaterial, cost-effective, and durable, and has already been approved for various medical devices. The high mechanical strength and low degradation rate of PCL make PCL-based biomaterials advantageous for hard tissue engineering applications, particularly for long-term implantation. PCL can be incorporated with different polymers and other inorganic materials. This strategy has been widely applied to design scaffolds with appropriate properties for bone and dental tissue engineering. The following two reports focus on printing PCL with a ceramic composite.

For dental tissue applications, high contents of the widely used ceramic biomaterial beta-TCP are required to mimic the chemical composition of bone minerals providing a good environment for osteogenesis and osseointegration. As described in the section ‘Materials as nanocomposites for printing and bioprinting’, PCL is a biodegradable polymer with a low glass transition temperature favoring great processing for 3D printing, while it degrades slower due to its longer hydrophobic aliphatic chains compared to other biodegradable polyesters. Hence, the use of blends of beta-TCP with PCL may prove effective for enhancing the bioactivity of 3D-printed bone-like scaffolds. Melt blends of beta-TCP and PCL were prepared with high beta-TCP contents of 50 and 70 wt%, and the composites were used to fabricate 3D scaffolds using an STL file and an extrusion layer-by-layer 3D printing system [182]. The scaffold composition with the higher beta-TCP content (70 wt%) displayed rougher morphology and higher porosity and improved cell proliferation, migration, and alkaline phosphatase activity. Regarding the mechanical properties, the composite scaffolds with the beta-TCP content of 70 wt% indicated lower compressive strength values compared to the ones with lower ceramic content and the pure PCL scaffolds due to the strong contribution of the brittle ceramic component. In another study, PCL has been combined with polydopamine-modified calcium silicate (PDACS) to fabricate 3D-printed composite scaffolds with Wharton’s jelly mesenchymal stem cells incorporated with human umbilical vein endothelial cell (HUVEC)-laden hydrogel [177]. Dopamine is an analogue of 3,4-dihydroxy-l-phenylalanine (DOPA), which has been recently applied for the modification of various substrates, due to its strong adhesive properties in mussels. For the formation of the composite construct, the cell-loaded hydrogel and the extruded PDACS/PCL were dispensed alternately in layers. The HUVECs in the bioink showed higher levels of angiogenic markers and capacity for the formation of vascular networks. Higher levels of bone formation markers were also determined. These hybrid composite materials with cells promote osteogenesis and stimulate angiogenesis, highlighting that 3D printing is an effective approach for the regeneration of damaged complex hard tissues, such as the teeth tissues. 

The application of hydroxyapatite–polymer composites based on 3D-printed grafts demands a thorough optimization of the mechanical and biological properties for their use as medical devices. Hydroxyapatite–zinc-functionalized starch composites have been fabricated by 3D extrusion printing as artificial bone graft substitutes [183] and optimized to predict the best composition. The compressive strength of the grafts was significantly improved by parametric optimization and functionalization with zinc ions. The functionalized grafts maintain their mechanical strength for six weeks in simulated body fluid, while the nonfunctionalized starch–HA grafts completely degrade in a week. The functionalization with zinc ions exhibited an antibacterial activity against S. aureus and increased osteoblastic cell viability. The optimization methodology may lead to time for experimentation and cost reduction in 3D printing, while it highlights the importance of understanding composition and process dependence for producing functionalized 3D-printed structures for low load-bearing implants that can be used in craniomaxillofacial bone.

Apart from the calcium-containing composites, zirconia-based composites are extensively used in dental applications due to their high mechanical strength. Acrylate ester-based dental resins were reinforced with different concentrations of zirconia nanoparticles. The effect of the zirconia nanoparticle volume fraction addition of the 3D-printed nanocomposites on the mechanical and physical properties was evaluated by aging in artificial saliva [178]. The results show that the nanohardness and elasticity behaved symmetrically with the maximum strength at a 3 wt% addition of zirconia nanoparticles, and this loading showed the highest fracture toughness and modulus. In addition, the improvement of flexural strength was proportional to filler concentration, and all properties were dramatically enhanced after three months of aging in artificial saliva, suggesting that these 3D-printed nanocomposites are promising as long-term provisional dental restoration materials.

Polymer-infiltrated ceramic network (PICN) composites are compatible with human enamel due to their mechanical attributes, and thus they are efficient dental restorative materials. Three-dimensional-printable PICN composite as a restorative material have been fabricated starting with the production of a 3D-printable precursor slurry with a high concentration of silica nanoparticles and 3D printing using stereolithography [179]. The 3D-printed object was sintered to create a nanoporous structure, infiltrated, and polymerized with a resin monomer. The 3D-printed PICN composites displayed a nanosized dual network structure of a silica backbone with infiltrated resin, had a similar Vickers hardness to enamel, and a similar elastic modulus to dentin, indicating comparable flexural strength to the CAD/CAM model.

Current processing strategies in ceramics have limitations to create objects with complex or customized geometries due to restrictions on the shape of mold. Ceramics with great toughness are needed for a variety of applications, such as dental restorations due to their outstanding chemical and mechanical stability. Digital light processing (DLP) is a fast-growing additive manufacturing technique able to produce high performance, defect-free objects with a good degree of precision and biomimetic toughening design [184]. Polymer infiltration was performed to obtain composites by slow impregnation of a commercial epoxy polymer into the as-sintered ceramics. The zirconia–epoxy composites were cured to ensure completion of the polymerization and polished with sandpaper. The fabricated ceramic composites display remarkable improvement in toughness compared to those of pure ceramics and have customized geometries, making them useful in dental restorations.

**Figure 3:**
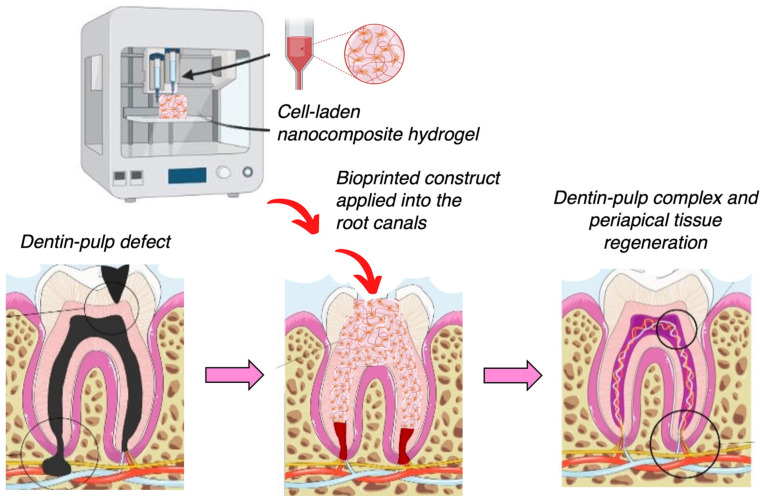
Bioprinting of cell-laden hydrogel constructs for the regeneration of dentin pulp (Modified image adapted with permission from [186], 2022, MDPI AG).

Apart from the dental bone grafts, the regeneration of tooth-like composite tissue is a challenging area of dental tissue engineering. The 3D bioprinting technology is a promising tool for the manufacturing of a dentin–pulp complex with patient-specific shapes and a homogenous distribution of cells within a hydrogel-based matrix. A fibrin-based bioink composed of fibrinogen, gelatin, hyaluronic acid, and glycerol was designed using CT data for bioprinting with the encapsulation of human dental pulp stem cells to produce tailor-made 3D constructs by inducing localized cell differentiation [185]. Different fibrinogen concentrations within the bioink have been evaluated, indicating great printability in micropatterning up to 160 μm without cell damage and high cell viability for 25 days in culture. The odontogenic differentiation of cells in the bioinks was regulated according to the fibrinogen concentration as it is affected by the physical properties, such as the stiffness of the bioink. By applying the bioinks to bioprinting, the outer dentin region of the printed construct promoted the odontogenic differentiation, whereas the central pulp region maintained undifferentiated. For the future, the printing process for surrounding tissues, including cementum and periodontal ligament tissues, should be considered in the development to regenerate a whole tooth. Figure 3 presents a schematic on the bioprinting of cell-laden hydrogel constructs for the regeneration of dentin pulp.

### 4.6. Bioprinting of Nanocomposites for Cardiovascular Applications

Biofabricated cardiac patches that mimic the myocardium extracellular matrix and enable integration with the host tissue offer a potential solution for myocardial infarction repair as they can be directly implanted on myocardial infarct [187]. However, the biofabrication of cardiac constructs is challenging due to their complex characteristics. They should be electrically conductive, elastic, mechanically strong, prevascularized, and biologically active. A method for 3D bioprinting cardiac constructs has been reported using a nanoreinforced hybrid patch based on ultraviolet (UV) irradiated methacrylated collagen (MeCol) loaded with human coronary artery endothelial cells [13]. The MeCol-cell constructs were functionalized with carbon nanotubes (CNTs) and alginate as a matrix. Their functionalization indicated a nanofibrous network with improved viscoelastic behavior, electrical conductivity, and cell adhesion and elongation. The CNT-reinforced 3D-printed hybrid constructs showed high stiffness and electrical conductivity, maintaining a high swelling ratio and cell response, presenting potential prevascularized cardiac patches. Another strategy of 3D bioprinting of a composite bioink has been described to fabricate an endothelialized myocardium-on-a-chip platform for cardiovascular toxicity evaluation. This was based on endothelial cells directly bioprinted within microfibrous hydrogels migrated to the periphery of the microfibers to form an endothelium-like layer [188], which was seeded with cardiomyocytes with controlled directionality as in myocardium tissue. The bioprinted constructs were embedded into a microfluidic perfusion bioreactor as a platform for drug screening. Furthermore, a method based on microfluidic bioprinting to generate a scaffold with multilayer hydrogel microfibers has been described using the coextrusion of two flows of a hybrid bioink composed of GelMA and alginate [189]. A microfibrous scaffold could be obtained by blending alginate with GelMA via ionic crosslinking in the presence of divalent ions and a further photocrosslinking of GelMA for increased stabilization. The endothelial cells encapsulated within the bioprinted microfibers were able to treat lumen-like structures similar to the vasculature to construct a vascularized tissue by seeding another cell type into the space of the microfibers.

Vascularization is a prerequisite for the survival of complex 3D-bioprinted tissue-engineered constructs as it is responsible for the sufficient supply of oxygen and nutrients to cells. In fact, the biofabrication of complex tissues in large sizes with random shapes with integrated prevascularization still faces many challenges. Composite scaffolds were fabricated from GelMA and carboxymethyl chitosan (CMCS) and combined with BM-MSCs to assess their angiogenic potential [190]. The composite GelMA/CMCS scaffolds indicated enhanced mechanical properties and vasculogenesis-related gene expression, while 3D-bioprinted GelMA/CMCS constructs showed their capacity for vascular tissue engineering. Another study reports on 3D-bioprinted hollow chitosan/hydroxyapatite fibers by combining a coaxial extrusion with a water-bath crosslinking [191]. Degradation indicated that the CS/HA scaffolds had a high potential to adapt to a human environment. The addition of an appropriate HA quantity increased the mechanical properties. Moreover, cell viability was high in the hollow constructs, indicating good in vitro biocompatibility. This study provides a method for the production of composite materials for vascularization by fabricating scaffolds with microchannels for tissue engineering applications.

Recently, a platform for coculturing human umbilical vein endothelial cells (HUVECs) and C2C12 myoblasts using cell electrospinning and 3D bioprinting has been described [192]. PCL and collagen patterns with a topographical motif were used as physical supporters for the deposition of HUVEC-laden alginate bioinks by cell electrospinning, a method that improves the cell–matrix interaction by embedding cells into nanofibers. The electrospun HUVECs showed high cell viability and growth, while myoblasts were cocultured on the HUVEC-laden fibers to facilitate myoblast regeneration. The constructs comprising myoblasts and HUVECs displayed enhanced myogenesis evidenced by the myogenic-specific gene expression compared to the scaffolds that included only myoblasts. The potential of 3D printing has been investigated to manufacture microfluidic channel networks of complex 3D tissues [193]. The reverse engineering software was used to design a CAD model. Poly(vinyl alcohol) (PVA) was used as sacrificial material to print a stent in one bioprinter nozzle, while in a second nozzle, a hydrogel composite of H9c2 and HUVECs was mixed with sodium alginate, agarose, and platelet-rich plasma as bioink and extruded to deposit on the internal pores of the sacrificial scaffold. The scaffold dissolved and changed to a complex microfluidic channel network, and cells retained their biological properties within the printed constructs.

Although stereolithography bioprinting is the primary technique for polymer resin bioinks, it lacks the ability to print multiple materials and cell types to include fillers and other biological components at specific locations in the scaffolds. In a recent study, bioinks from a polymer resin PEGDA were used to fabricate gradient scaffolds for complex tissue engineering by extrusion bioprinting [194]. Bioinks were prepared by adding cellulose nanocrystals (CNCs) into PEGDA at ratios that reach the viscosity needed for extrusion printing. The bioinks were printed into single- and multiple-material (gradient) scaffolds using a commercial bioprinter and crosslinked with a photoinitiator (lithium phenyl-2,4,6-trimethylbenzoylphosphinate). Some CNC composition displayed brittleness after crosslinking and were thus not suitable, while other bioink compositions were used to create gradient scaffolds with great mechanical properties. The results show that the extrusion bioprinting improves the reliability and functionality of the scaffolds. The presence of perfusable vascular systems is essential for the fabrication of thick tissues that can function like native tissues. Bioprinting a hydrogel-based 3D vasculature-like structure in a single step is a big challenge. To this end, a hydrogel-based composite fabricated from Pluronic 127 and GelMA that offers great printability, shape integrity, and biocompatibility for 3D bioprinting of a perfusable complex vasculature-like structure has been investigated [195]. The hydrogel composite is printable at human body temperature, while it supports the cell proliferation of fibroblasts and HUVECs and cell differentiation, representing a new vascularization strategy for 3D bioprinting in tissue engineering. In another study, the photocrosslinkable hydrogel precursors GelMA and PEGDA were combined with alginate to produce a blend for extrusion 3D bioprinting. The parameters associated with photocrosslinking hydrogels including the photoinitiator type and concentration, and light intensity were assessed for their effect on the embedded cells in the bioprintable extrusion [196]. Irgacure was found to increase the hydrogel stiffness with a proportional decrease in cell viability compared to VA086 in the 3D cell–hydrogel culture. Human AD-MSCs survived increasing photoinitiator concentrations under the bioprinting conditions better than other cell types, such as aortic valve interstitial and smooth muscle cells. This study indicates various parameters that optimize cell viability during 3D printing for different cell types revealing that oxidative stress is higher in photocrosslinking conditions leading to cell viability reduction.

Another interesting aspect has been reported on the evaluation of a 3D bioprinting technology to fabricate scaffolds for endothelial cell repair [197]. Different PEG, PLA, and pluronic F127 compositions were optimized to produce viscous bioinks, while dimethyloxalylglycine and erythropoietin have been used as model drugs to load the inks. The bioinks revealed a homogenous distribution of the materials, and the drug release was sufficient to increase HIF-1 alpha expression, while increased expression of the VEGF gene was after 30 min. These results prove that bioprinting is suitable for fabricating composite scaffolds with potential applications in endothelial cell repair in cardiovascular disease. Another application of bioprinting reports on engineered nonviral gene-activated constructs reinforced by polymeric microfilaments using RGD-gamma-irradiated alginate and HAp complexed to plasmid DNA [198]. BM-MSCs were encapsulated within the ink and bioprinted with a PCL supporting material for mechanical stability. Therapeutic genes encoding for BMP and TGF were delivered to promote osteogenesis and matrix deposition following the incorporation of plasmid DNA. The subcutaneous implantation of gene-activated cell-laden constructs displayed high levels of vascularization and mineralization, validating their use in biological functionality.

**Figure 4:**
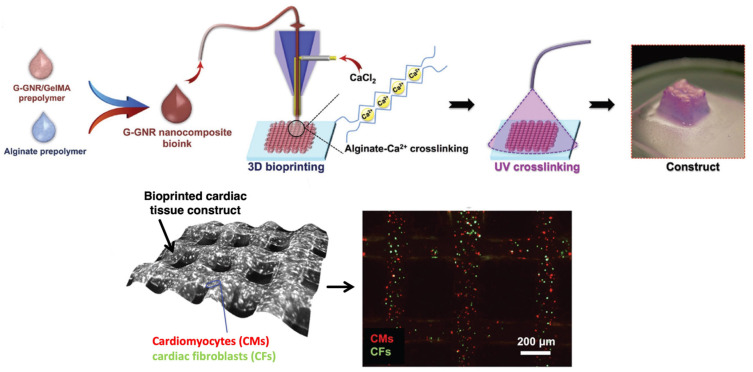
Schematic showing 3D bioprinting using GelMA-coated gold nanorod (G-GNR) nanocomposite bioink. Bright field image shows homogeneously distributed cardiomyocytes and cardiac fibroblasts in bioprinted cardiac tissue construct. Adapted with permission from [114], 2022, Wiley VCH Verlag GmbH & Co KGAA.

In summary, it has been shown that effective vascularization is essential for the survival and functionality of artificial complex tissue-engineered organs. Although the formation of vascular networks from endothelial cells have been used in engineered organs, recent studies using different cell populations focus on multicellular interactions with endothelial cells, revealing that cocultures present a more effective strategy in organ revascularization. In addition, the use of appropriate printing techniques and the selection of bioreactor systems are crucial. Figure 4 presents a schematic of 3D bioprinting using a GelMA-coated gold nanorod with alginate nanocomposite bioink for the fabrication of a bioprinted cardiac tissue construct.

### 4.7. Advances of Nanocomposite Bioprinting for Organ-on-a-Chip and Biosensor Applications

Organ physiology encompasses many individual intricate biological systems, often making the development of new regenerative medicine strategies quite challenging, as they have to adopt a more holistic perspective to cope with the requirements that the precise medicine imposes. The organ-on-a-chip technology is a relatively new field based on the combination of various biological, chemical, and engineering advancements in order to produce miniature-like structures that resemble the microenvironment of the native tissue [199,200]. Its main concept revolves around the integration of different microfluidic chips as parts of polymeric constructs that can manipulate cell behavior at close proximity and thus mimic small-scale physiological processes. Because the main ingredient of microchips is silicon, organ-on-a-chip structures can be considered as nanocomposites.

Three-dimensional bioprinting is an excellent method for the construction of organ-on-a-chip platforms, as it can enable the printing of heterogeneous 3D structures with great spatial resolution. So far, the intermixing of these two techniques has found application in the assembly of different artificial organ types, such as cardiac [188], bone [201], hepatic [202], lung [203], and vascular [204] tissues. In a recent report, a vessel-on-a-chip was created by 3D bioprinting a GelMA-based bioink containing endothelial and smooth muscle cells on a microfluidic chip consisting of poly(methyl methacrylate) (PMMA) [205]. This system exhibited great cell viability and proliferation, while the presence of the microchip and the coculture led to an upregulation of proteins expressed specifically by these two cell types.

Biosensors are devices based on biological, chemical, and electromechanical principles, with the purpose of detecting biochemical fluctuations inside the human body, such as changes in the levels of various physiological biomolecules or even identify markers that are connected with specific pathophysiologies [206]. They usually consist of three different subsystems: (i) a receptor and detector that can capture the examined biological process, (ii) a transducer that can transmute the biological information to another processable form, such as light or electrical current, and (iii) a monitoring system that can collect the information and present it with quantitative optical cues [207,208].

Biosensing and 3D bioprinting techniques have been explored in conjunction with both tissue engineering [123] and biosensing applications [209]. By using the inkjet bioprinting method, a nanocomposite bioink was prepared by depositing C2C12 myoblasts following a specific alignment pattern onto small silicon-based cantilevers [210]. After 4 days in culture, the 3D-bioprinted devices were subjected to a series of electrical pulses at a standard voltage and time period, but with alternating frequencies, ranging between 1 and 10 Hz, to examine the contractility of the myoblasts. The silicon cantilevers responded excellently to the exogenous stimulation signal, with the cells exhibiting the expected contractility pattern, while retaining high levels of biocompatibility.

## 5. Conclusions

In summary, the field of nanocomposite 3D bioprinting holds high promise for the development of novel multifaceted materials with tremendous therapeutic potential in virtually any tissue engineering discipline. Biomaterials derived from natural, natural/synthetic, or purely synthetic sources can be designed in a variety of configurations for the highly controlled development of active nanocomposite bioinks. Nanoparticle-based additives and other modifications explored for the development of composite bioinks have been shown to significantly enhance rheological, biological, and structural material properties. The inclusion of nanoparticles in composite bioinks provides several major advantages including the ability to deliver niche biological cues, serve pertinent therapeutic functions, as well as aid in the physicochemical and printing properties of scaffolds. Some of the challenges of employing nanoparticles and other additive manufacturing techniques include the optimization of temperature, speed, and pressure in alignment with that of the additional materials, such as live cells, included in the bulk bioink to produce structures of homogenous resolution. This is becoming more successful with the variety of bioprinting techniques, such as inkjet, laser-assisted, extrusion and multinozzle printing. The application of such materials has been and continues to be widely researched for bone, cartilage, dental, and cardiovascular tissue engineering purposes. The extent of material modification made available by the use of such nanobased additives opens many doors for preclinical and clinical applications in the future.

## Figures and Tables

**Table 1:** A brief overview of the major bioprinting techniques with their advantages and disadvantages.

3D Bioprinting Method	Physical Principle	Advantages	Disadvantages	References
Extrusion-based 3D bioprinting	The most conventional 3D bioprinting technique, based on the use of varying pressure and temperature values to formulate bioprinted constructs of hierarchical architecture.	Continuous extrusion reinforces the robustness of the scaffolds; Tackles viscosity issues more efficaciously.	High stresses and temperatures developed during fabrication can adversely affect cell viability.	[21,127,129]
Inkjet 3D bioprinting	A method that does not require direct contact, utilizing piezoelectric, thermal, and electromagnetic sources in order to direct the ejection of multiple bioink droplets into different 3D shapes.	High printing fidelity; High printing velocity; Facilitates the miscibility of different biomolecules; Insubstantial effect on cells viability during printing.	Only low-viscosity bioinks are printable.	[28,132,133]
Stereolithography 3D bioprinting	A technique that relies on the crosslinking of a photopolymerizable bioink solution, after its pouring into a mold with desired geometrical properties and its solidification under the irradiation from either a laser or UV light source.	High spatial resolution; Use of predesigned molds enhances printing fidelity.	Slow process because it consists of two phases, UV and laser irradiation can damage cells; The dispersed nanophase can affect the extent of photopolymerization due to light scattering.	[6,27]
Laser-assisted 3D bioprinting	A laser beam is guided toward sequential bioink droplets, resulting in heating them and, eventually, leading to their deposition on a surface, without requiring direct contact with this target area.	Fast printing speed. Allows for high initial cell number entrapment in the bioink droplet.	Laser beam can potentially harm cells due to the heat absorbed by the droplet.	[27,65,138]

## Data Availability

The data presented in this study are available on request from the corresponding author.
